# Supplementary material for: Rift linkage and inheritance determine collisional mountain belt evolution
Source: Nat Commun. 2025 Dec 4;17:84. doi: 10.1038/s41467-025-66695-8 (PMC12770315; doi:10.1038/s41467-025-66695-8)
Supplement: Supplementary file 1 — Supplementary Information [file 41467_2025_66695_MOESM1_ESM.pdf]

Supplementary Information for: Rift linkage and inheritance determine collisional mountain belt evolution

Sebastian G. Wolf[1*], Ritske S. Huismans[1], Josep Anton Muñoz[2], Dave A. May[3]

[1] *Department of Earth Science,University of Bergen, Bergen, Norway*
[2] *Geomodels Research Institute, University of Barcelona, Barcelona, Spain*
[3] *Institute of Geophysics and Planetary Physics, Scripps Institution of Oceanography, University of California San Diego, La Jolla, CA, USA*

Content

This supplementary file contains:

1. An explanation of the supplementary model animations.
2. Supplementary plots of Models 1 - 4 showing the deformational regime and deviatoric stresses.
3. Three supplementary models with description
4. Background information on the force balance analysis
5. The table with mechanical and thermal properties of model materials

1 Model Animations

The data repository contains 6 animations of the main end-member models M1-M4 and the supplementary models SM1 and SM2. The models are displayed in 3D-view with the thermo-mechanical model as slices on the right side, and the landscape evolution model on the left. The slices of the thermo-mechanical model show material colours (see legend in Fig. 9) with temperature contours (100 °C, 350 °C, 550 °C, 1330 °C), and strain-weakened areas as dark grey overlay.

[*] Corresponding Author, e-mail: sebastian.wolf@uib.no

2 Deformational regimes and deviatoric stresses of Models 1 - 4

Figures S1 - S4 show cross sections of the deformational regimes and deviatoric stresses of the four main models presented in the manuscript. Note that the fields were computed during post-processing, with one average value per cell. In pTatin3D, properties are computed at each Lagrangian marker particle and then averaged over each cell. Because of this averaging, we expect very small differences between the fields presented here and the fields used for computation in pTatin3D.

3 Supplementary models

Supplementary Model 1 (SM1, Fig. S5) has the same boundary and initial conditions as Model 1, with the difference of a small seed offset of 20 km. This model has a very similar evolution as Model 1. Extension creates a linked and slightly offset rift basin (Fig. S5a). Inversion retains the rift structure and forms a continuous small mountain belt with river flow perpendicular to the orogen boundary (Fig. S5b). Further growth links the two west-dipping faults of the previous rift basins, leading to same-polarity subduction of the lower crust and lithospheric mantle. Growth continuously retains the initial basin configuration with minor changes along strike (Fig. S5c).

Supplementary Model 2 (SM2, Fig. S6) has the same boundary and initial conditions as Model 3, with the difference of a smaller seed offset of 100 km instead of 200 km. This model has a very similar evolution as Model 2. Extension creates a linked and offset rift basin (Fig. S6a). Initial inversion retains the rift structure, forming a continuous small mountain belt with river flow perpendicular to the orogen boundary (Fig. S6b). Continuous shortening and growth is accommodated by opposite polarity subduction, and reversal of the inherited rift structure, forming a characteristic topographic low in the transitional domain (Fig. S6c).

Supplementary Model 3 (SM3, Fig. S7) has the same boundary and initial conditions as Model 4, but an opposite polarity in dip of the weak seeds. This model has a very similar evolution as Model 3. Extension creates a linked offset rift system with asymmetric margins depending on seed dip (Fig. S7a). Inversion retains the rift structure and forms a continuous small mountain belt with river flow perpendicular to the orogen boundary (Fig. S7b). Further growth links the inner faults of the previous rift basins related to the initial weak seeds, leading to opposite polarity subduction of the lower crust and lithospheric mantle. Growth continuously reverts the extensional structure and creates discontinuous topography with two high mountain belts related to the inverted rift basins and a topographic low in the transition zone (Fig. S7c).

4 Force balance analysis

The following section provides more background information on the force balance as described in the main text. We introduce the depth-integrated strength of the lithosphere $F_{int} = \int_{LAB}^{surface} \tau \, dz$, where τ is the maximum deviatoric flow stress. In case the lithosphere has no pre-existing weaknesses $F_{int} = F_{strong}$, if the lithosphere is fully strain-weakened $F_{int} = F_{weak}$ (see Fig. 6a). Assuming lithostatic pressure conditions, a constant typical strain rate of $1 \times 10^{-14}\,\mathrm{s}^{-1}$, and a steady-state thermal field corresponding to the initial thermal field of the models gives $F_{weak} = 2.8 \times 10^{12}\,\mathrm{N/m}$ and $F_{strong} = 12.1 \times 10^{12}\,\mathrm{N/m}$. These assumptions neglect tectonic overpressure, e.g. related to gravitational potential energy variability or far-field deformation, which can be significant[1,2] and is also observed in our models (Figs. S1 - S4). Sensitivity tests including substantial overpressure showed no first-order effects on the resulting relationships for basin linkage or subduction polarity, but would increase F_{weak} and F_{strong}. These tests suggest that the analytical results are robust within the framework of our assumptions, and show that the reported values of F_{weak} and F_{strong} are minimum values.

Concerning basin-linkage: The force required for basin linkage can in most general terms be described as a sum of line-integrals:

$$F_{Link} = \int_{P_2}^{P_3} F_{int} \, dl + \int_{P_3}^{P_4} F_{int} \, dl \approx D' \cdot F_{strong} + D_{seed} \cdot F_{weak}. \tag{S1}$$

Equally, the force required for deformation to progress to the boundary can in general terms be described by:

$$F_{NoLink} = \int_{P_2}^{P_b} F_{int} \, dl \approx D_b \cdot F_{strong}. \tag{S2}$$

Whenever $F_{Link} < F_{NoLink}$, the basins link, and vice versa. This relationship also highlights that if F_{weak} was smaller, linkage would occur at larger basin offsets. A smaller F_{weak} could be related to very weak inherited weaknesses, and could be modelled with weaker and larger initial weak seeds.

Concerning subduction polarity: The surplus of deforming L_{IO} over L_{II} bears a penalty P that can be described as:

$$P = \int_{P_{O1}}^{P_{I2}} F_{int} \, dl - \int_{P_{I1}}^{P_{I2}} F_{int} \, dl \approx L_{IO} \cdot F_{weak} - L_{II} \cdot F_{weak}. \tag{S3}$$

Here, F_{int} corresponds to first order to the weak integrated strength F_{weak}, as the pre-collisional extensional structures are reactivated. Linking the same-dipping faults through the segment L_{IO} is only mechanically favourable if there is a net gain $G > P$. One such gain is for instance when the left-dipping shear zone with length L_{weak} in Fig. 6b is weaker than its conjugate right-dipping shear zone. In that instance we can express the gain as

$$G \approx L_{weak} \cdot F_{weaker} - L_{weak} \cdot F_{stronger}. \tag{S4}$$

References

1. Petrini, K. & Podladchikov, Y. Lithospheric pressure-depth relationship in compressive regions of thickened crust. *Journal of Metamorphic Geology* **18.** 290eh Times Cited:163 Cited References Count:69, 67–77 (2000).
2. Schmalholz, S. M., Medvedev, S., Lechmann, S. M. & Podladchikov, Y. Relationship between tectonic overpressure, deviatoric stress, driving force, isostasy and gravitational potential energy. *Geophysical Journal International* **197,** 680–696 (2014).
3. Gleason, G. C. & Tullis, J. A Flow Law for Dislocation Creep of Quartz Aggregates Determined with the Molten-Salt Cell. *Tectonophysics* **247,** 1–23 (1995).
4. Mackwell, S. J., Zimmerman, M. E. & Kohlstedt, D. L. High-temperature deformation of dry diabase with application to tectonics on Venus. *Journal of Geophysical Research-Solid Earth* **103,** 975–984 (1998).
5. Karato, S. & Wu, P. Rheology of the Upper Mantle - a Synthesis. *Science* **260,** 771–778 (1993).

Supplementary Table 1 | Mechanical and thermal properties of model materials.

**Parameters**	Sediments, Upper & middle crust	Lower crust	Mantle lithosphere	Sub-lithospheric mantle
Plastic rheology				
C - C_{sw} (MPa)	20-20	20-20	20-20	20-20
ϕ - ϕ_{sw} (°)	15-2	15-2	15-2	15-2
Viscous rheology				
Flow law[a]	WQtz	DMD	WOl	WOl
f	0.5	1	10	1
A ($Pas^{1/n}$)[b]	8.57×10^{-28}	5.78×10^{-27}	1.39×10^{-14}	1.39×10^{-14}
n	4.0	4.7	3.0	3.0
Q ($kJmol^{-1}$)	223	485	430	430
V (cm^3mol^{-1})	0	0	15×10^{-6}	15×10^{-6}
Density				
ρ_0 (kgm^{-3})	2800	2950	3365	3380
α (K^{-1})	3×10^{-5}	3×10^{-5}	3×10^{-5}	3×10^{-5}
Thermal				
k ($Wm^{-1}K^{-1}$)[c]	2.25	2.25	2.25	2.25
H (μWm^{-3})	1.15	0.5	0	0
c_p ($Jkg^{-1}K^{-1}$)	750	750	1250	1250

[a] WQtz is the wet quartz flow law as described in Gleason & Tullis [3]; DMD is the dry Maryland flow law based on Mackwell *et al.* [4]; WOl is the wet olivine flow law based on Karato & Wu [5].
[b] The laboratory derived pre-exponential flow law constant has been converted to conform with the second invariants of the stress and strain rates used in the model approach.
[c] Thermal conductivity for low temperatures. Between 1330 °C and 1340 °C the conductivity linearly increases from 2.25 to 52.0 $Wm^{-1}K^{-1}$, to mimic active mantle convection at high Nusselt number, keep the adiabatic gradient and prevent the system from cooling.

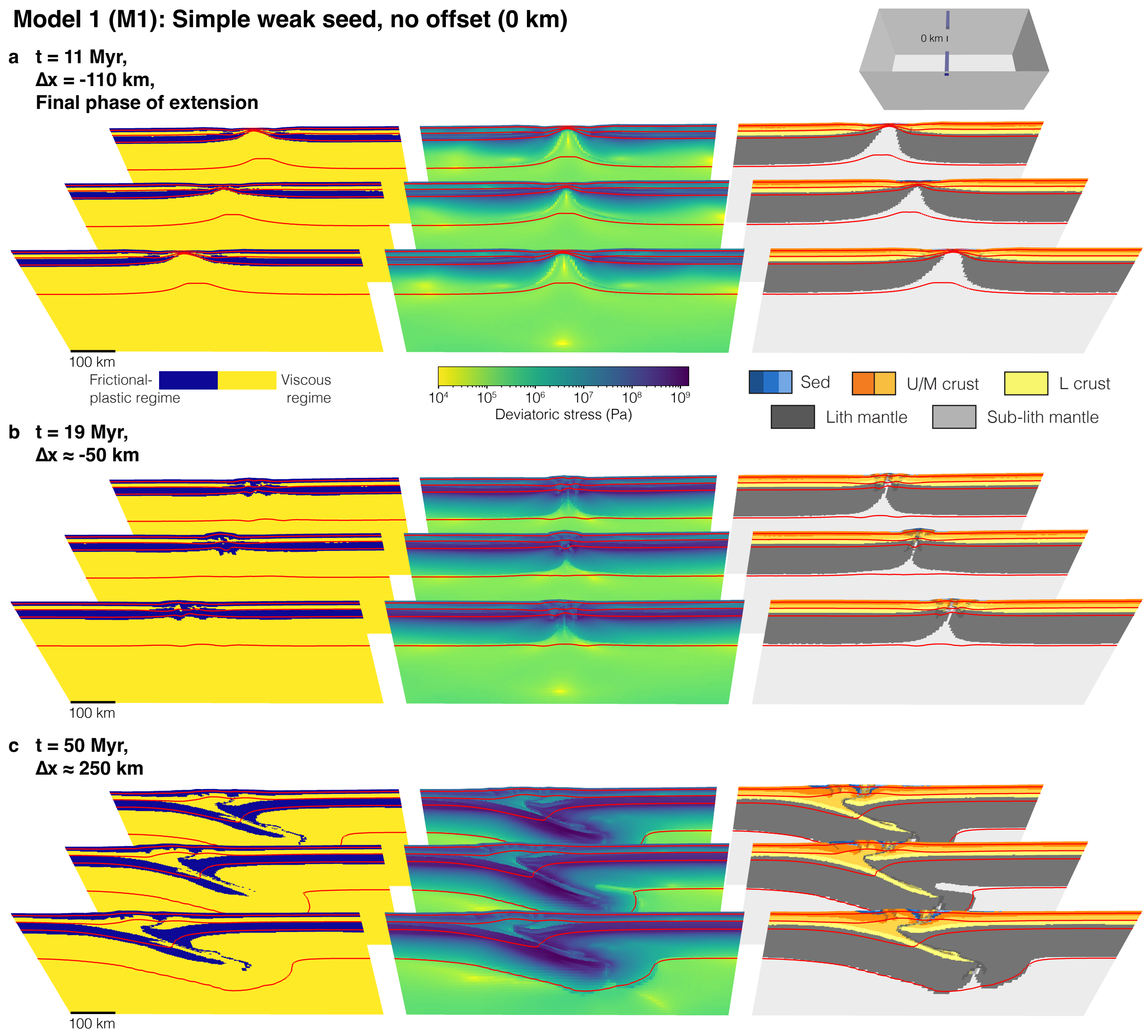

Figure S1 | Model 1 with 0 km offset and simple seeds: a) Snapshot at final stage of the rifting phase, b) Snapshot during the initial inversion stage, c) Snapshot during the mature mountain building phase. The small inset in the upper right corner shows the initial weak-seed configuration of the model. Each sub-figure shows three fields as cross-sections. The position of the cross-sections is equal to the position shown in the main model Figure (Fig. 2). Left cross-sections show deformational regime, highlighting where deformation would either be frictional-plastic or viscous. Middle cross-sections show deviatoric stresses. Right cross-sections show model materials. Abbreviations for model materials are as follows: Sed are sediments deposited during runtime; U/M crust or L crust are Upper/Middle and Lower crust, respectively; (Sub-)Lith mantle are the lithospheric and sub-lithospheric mantle. Red lines are temperature contours at 100 °C, 350 °C, 550 °C, and 1330 °C. Strain-weakened fault zones in the model materials cross sections are indicated by a grey semi-transparent overlay.

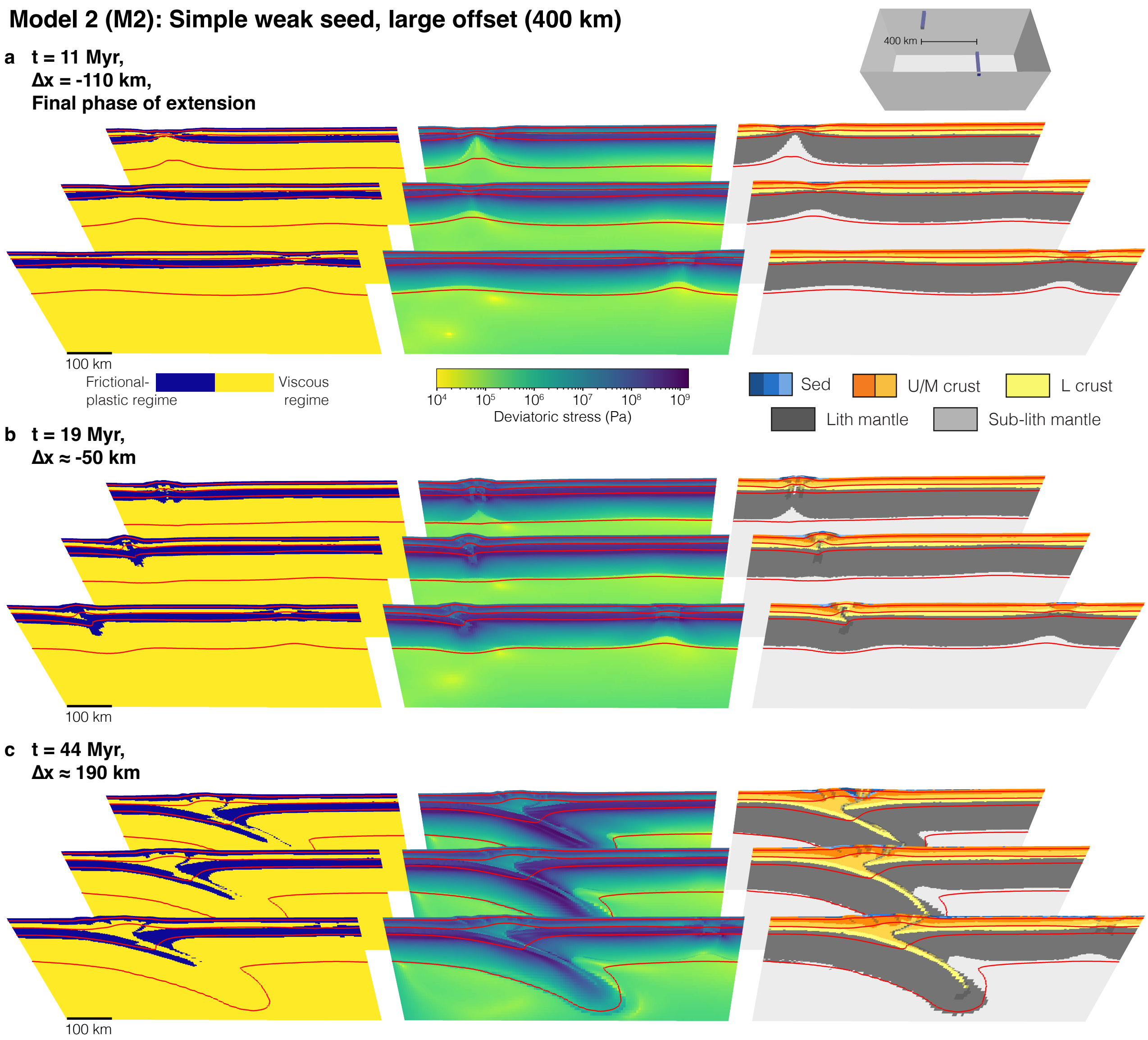

Figure S2 | Model 2 with 400 km offset and simple seeds: a) Snapshot at final stage of the rifting phase, b) Snapshot during the initial inversion stage, c) Snapshot during the mature mountain building phase. The small inset in the upper right corner shows the initial weak-seed configuration of the model. Each sub-figure shows three fields as cross-sections. The position of the cross-sections is equal to the position shown in the main model Figure (Fig. 3). Left cross-sections show deformational regime, highlighting where deformation would either be frictional-plastic or viscous. Middle cross-sections show deviatoric stresses. Right cross-sections show model materials. Abbreviations for model materials are as follows: Sed are sediments deposited during runtime; U/M crust or L crust are Upper/Middle and Lower crust, respectively; (Sub-)Lith mantle are the lithospheric and sub-lithospheric mantle. Red lines are temperature contours at 100 °C, 350 °C, 550 °C, and 1330 °C. Strain-weakened fault zones in the model materials cross sections are indicated by a grey semi-transparent overlay.

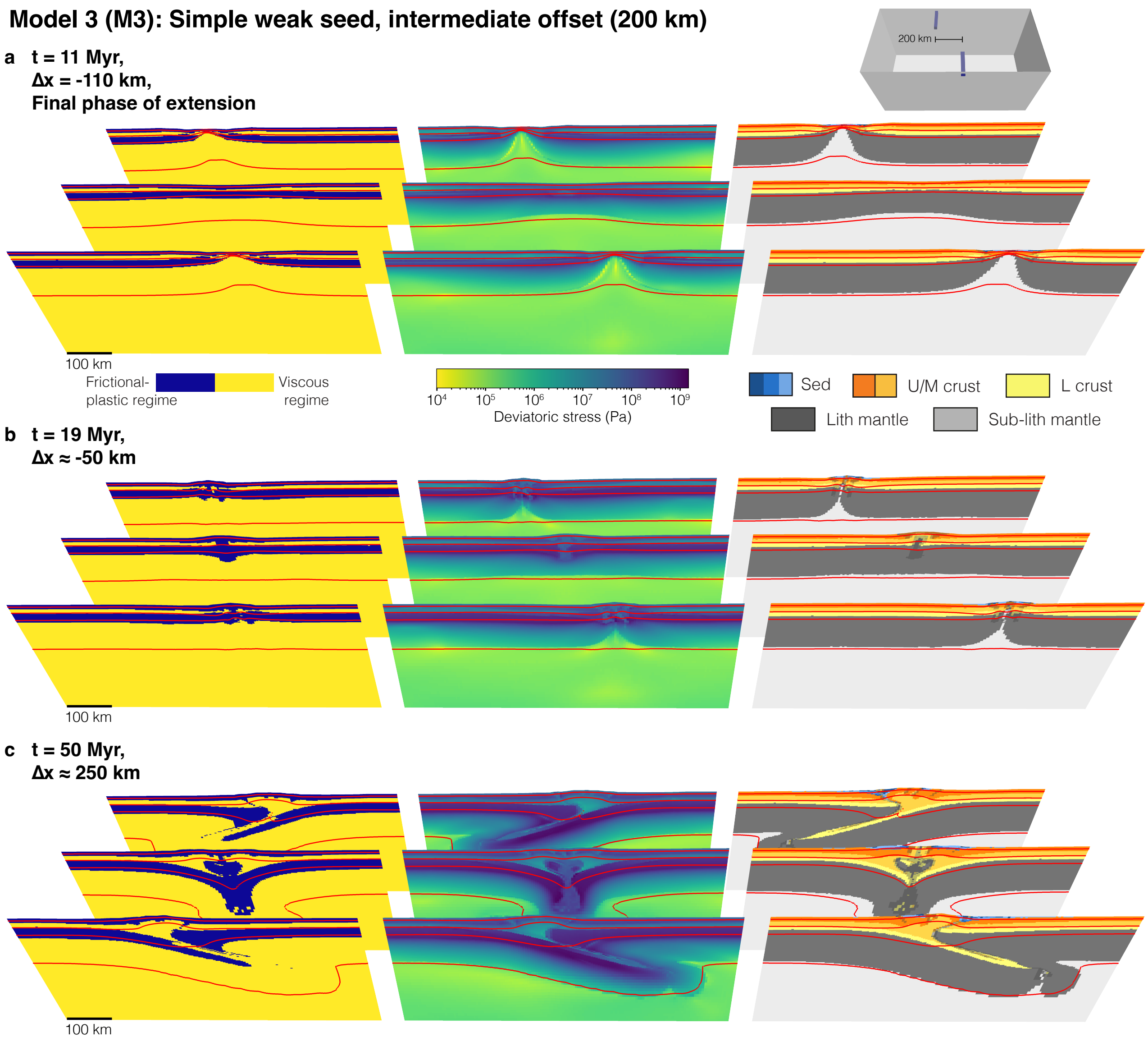

Figure S3 | Model 3 with 200 km offset and simple seeds: a) Snapshot at final stage of the rifting phase, b) Snapshot during the initial inversion stage, c) Snapshot during the mature mountain building phase. The small inset in the upper right corner shows the initial weak-seed configuration of the model. Each sub-figure shows three fields as cross-sections. The position of the cross-sections is equal to the position shown in the main model Figure (Fig. 4). Left cross-sections show deformational regime, highlighting where deformation would either be frictional-plastic or viscous. Middle cross-sections show deviatoric stresses. Right cross-sections show model materials. Abbreviations for model materials are as follows: Sed are sediments deposited during runtime; U/M crust or L crust are Upper/Middle and Lower crust, respectively; (Sub-)Lith mantle are the lithospheric and sub-lithospheric mantle. Red lines are temperature contours at 100 °C, 350 °C, 550 °C, and 1330 °C. Strain-weakened fault zones in the model materials cross sections are indicated by a grey semi-transparent overlay.

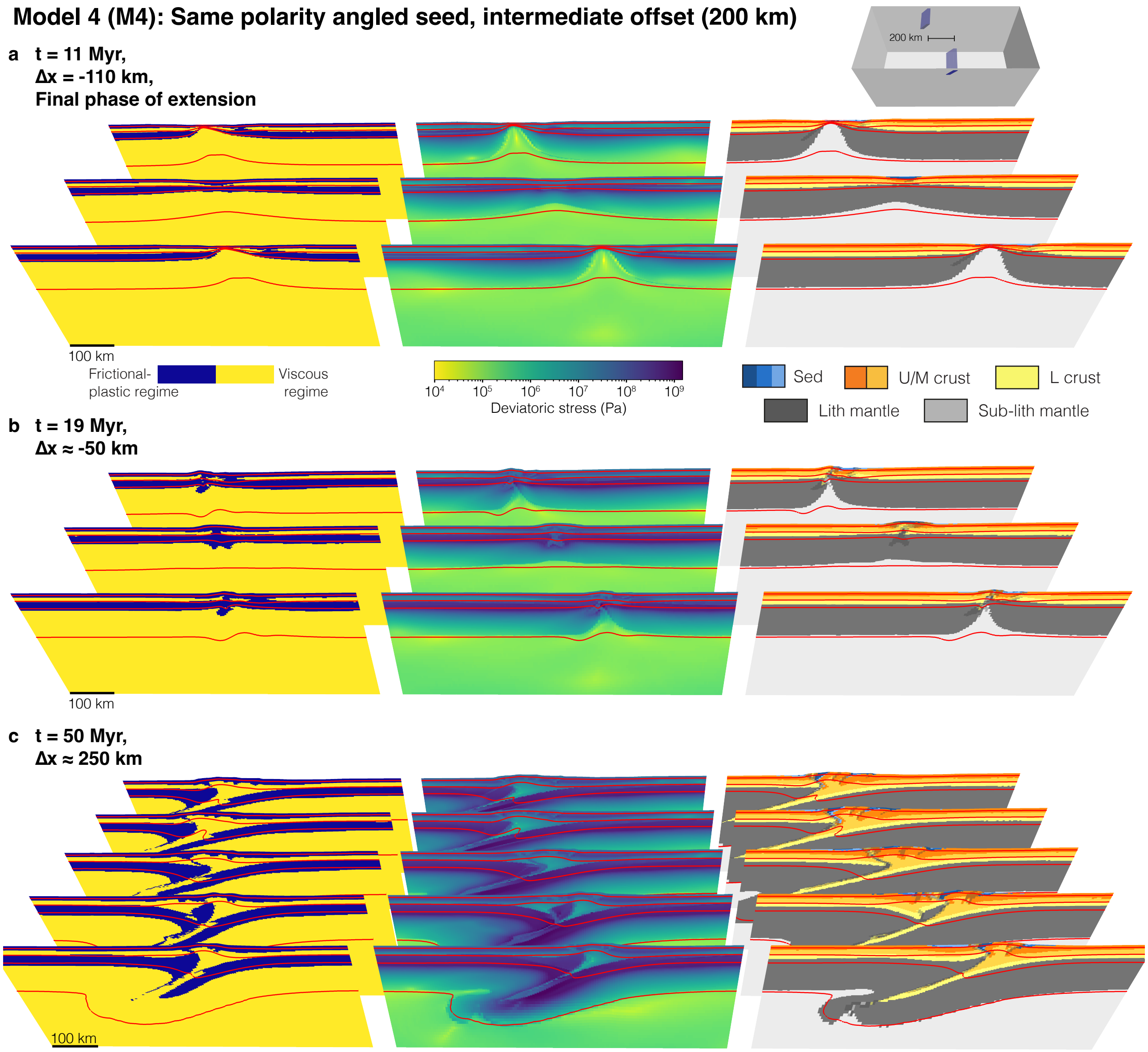

Figure S4 | Model 4 with 200 km offset and same-polarity angled seeds: a) Snapshot at final stage of the rifting phase, b) Snapshot during the initial inversion stage, c) Snapshot during the mature mountain building phase. The small inset in the upper right corner shows the initial weak-seed configuration of the model. Each sub-figure shows three fields as cross-sections. The position of the cross-sections is equal to the position shown in the main model Figure (Fig. 5). Left cross-sections show deformational regime, highlighting where deformation would either be frictional-plastic or viscous. Middle cross-sections show deviatoric stresses. Right cross-sections show model materials. Abbreviations for model materials are as follows: Sed are sediments deposited during runtime; U/M crust or L crust are Upper/Middle and Lower crust, respectively; (Sub-)Lith mantle are the lithospheric and sub-lithospheric mantle. Red lines are temperature contours at 100 °C, 350 °C, 550 °C, and 1330 °C. Strain-weakened fault zones in the model materials cross sections are indicated by a grey semi-transparent overlay.

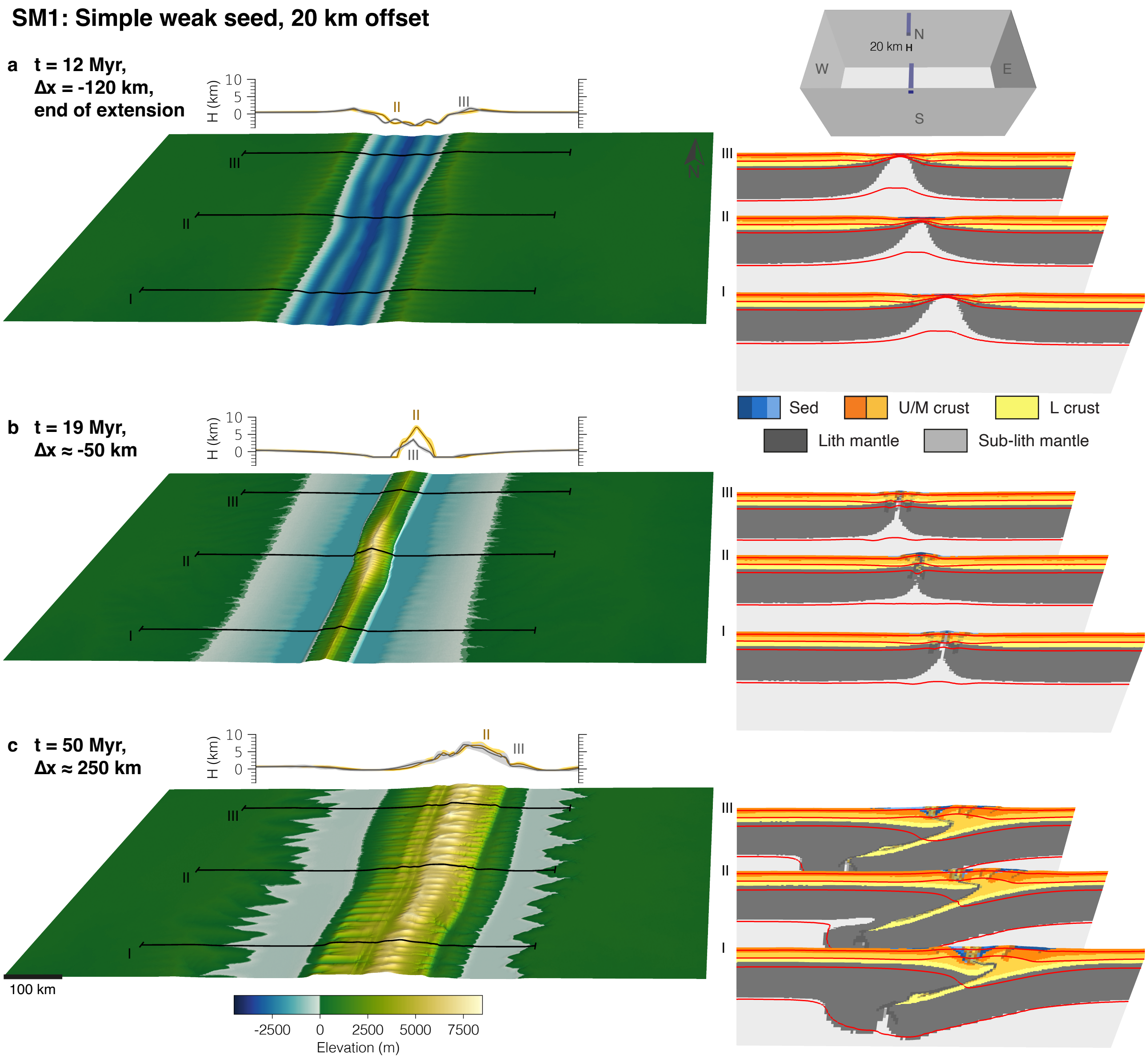

Figure S5 | Supplementary Model 1 with 20 km offset and simple seeds: a) Snapshot at the end of the rifting phase, b) Snapshot during the initial inversion stage, c) Snapshot during the mature mountain building phase. The small inset in the upper right corner shows the initial weak-seed configuration of the model with simple uniform seeds with 20 km offset. Each sub-figure consists of the model surface to the left with three corresponding cross-section shown to the right. Behind the model surface, two elevation-swath profiles at B and C are shown. Abbreviations for model materials are as follows: Sed are sediments deposited during runtime; U/M crust or L crust are Upper/Middle and Lower crust, respectively; (Sub-)Lith mantle are the lithospheric and sub-lithospheric mantle. Red lines are temperature contours at 100 °C, 350 °C, 550 °C, and 1330 °C. Strain-weakened fault zones in the model cross sections are indicated by a grey semi-transparent overlay.

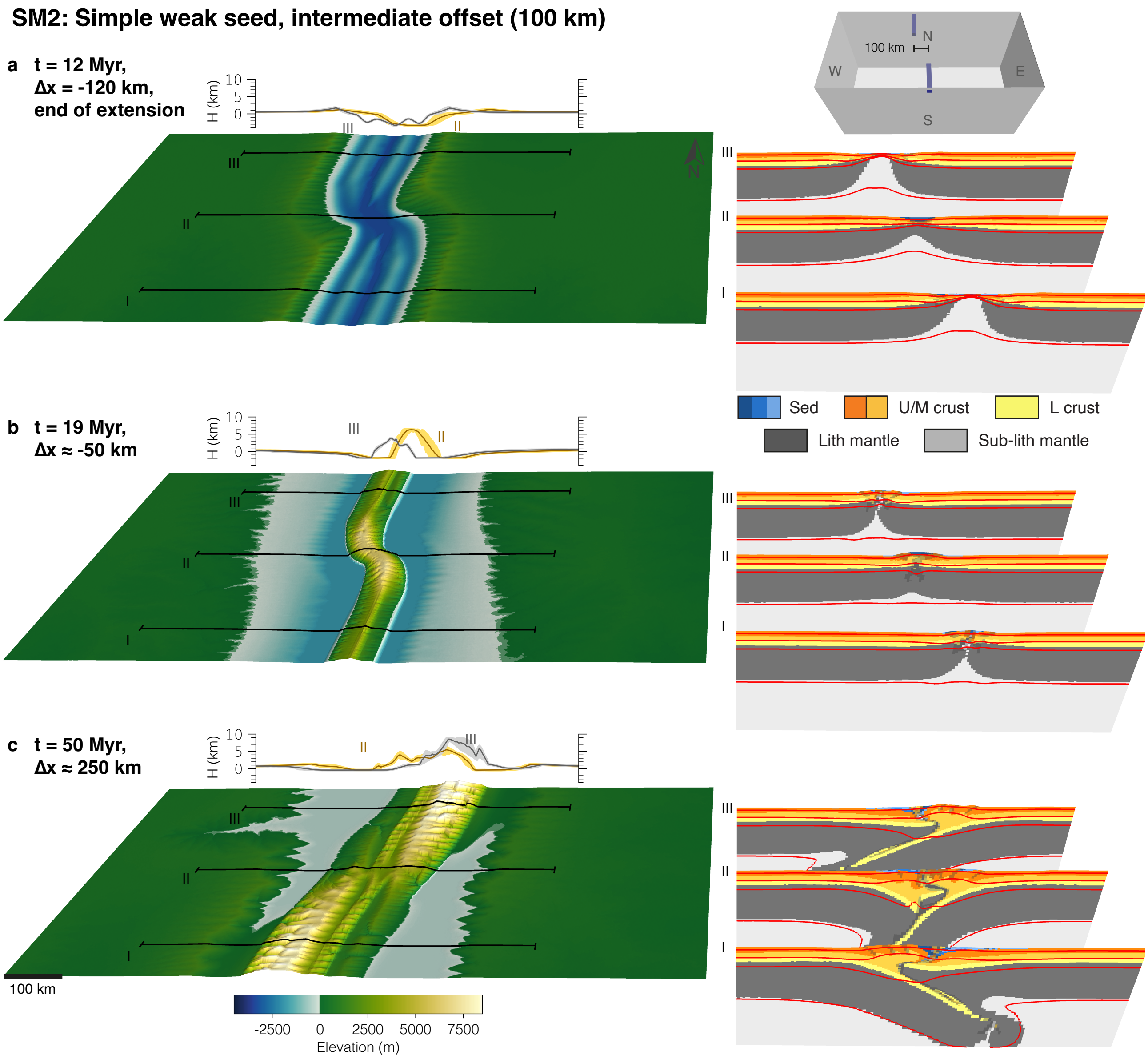

Figure S6 | Supplementary Model 2 with 100 km offset and simple seeds: a) Snapshot at the end of the rifting phase, b) Snapshot during the initial inversion stage, c) Snapshot during the mature mountain building phase. The small inset in the upper right corner shows the initial weak-seed configuration of the model with simple uniform seeds with 100 km offset. Each sub-figure consists of the model surface to the left with three corresponding cross-sections shown to the right. Behind the model surface, two elevation-swath profiles at B and C are shown. Abbreviations for model materials are as follows: Sed are sediments deposited during runtime; U/M crust or L crust are Upper/Middle and Lower crust, respectively; (Sub-)Lith mantle are the lithospheric and sub-lithospheric mantle. Red lines are temperature contours at 100 °C, 350 °C, 550 °C, and 1330 °C. Strain-weakened fault zones in the model cross sections are indicated by a grey semi-transparent overlay.

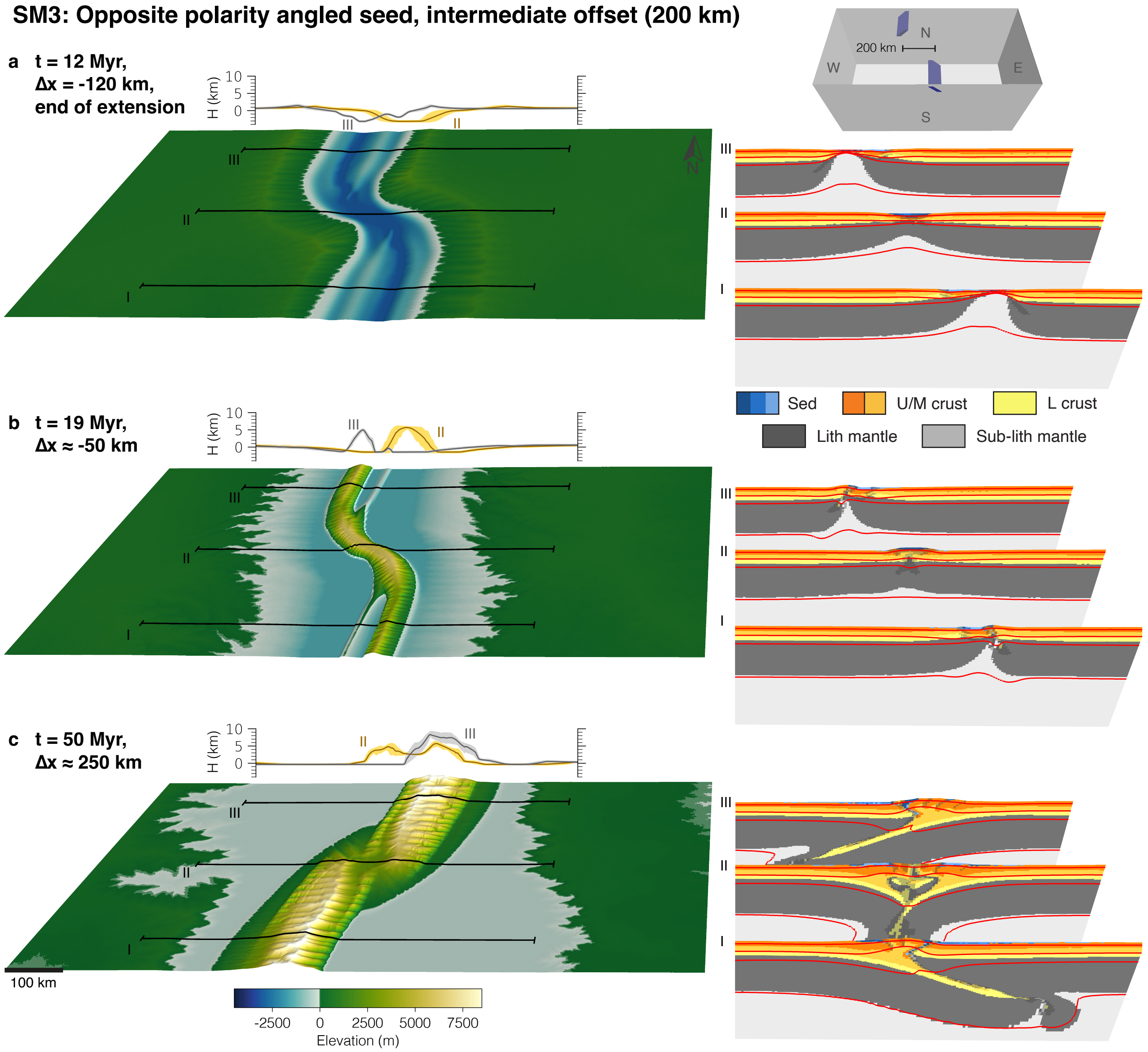

Figure S7 | Supplementary Model 3 with 200 km offset and opposite-polarity angled seeds: a) Snapshot at the end of the rifting phase, b) Snapshot during the initial inversion stage, c) Snapshot during the mature mountain building phase. The small inset in the upper right corner shows the initial weak-seed configuration of the model with oppositely dipping angled seeds with 200 km offset. Each sub-figure consists of the model surface to the left with three corresponding cross-section shown to the right. Behind the model surface, two elevation-swath profiles at B and C are shown. Abbreviations for model materials are as follows: Sed are sediments deposited during runtime; U/M crust or L crust are Upper/Middle and Lower crust, respectively; (Sub-)Lith mantle are the lithospheric and sub-lithospheric mantle. Red lines are temperature contours at 100 °C, 350 °C, 550 °C, and 1330 °C. Strain-weakened fault zones in the model cross sections are indicated by a grey semi-transparent overlay.
