# Supplementary material for: Rift linkage and inheritance determine collisional mountain belt evolution
Source: Nat Commun. 2025 Dec 4;17:84. doi: 10.1038/s41467-025-66695-8 (PMC12770315; doi:10.1038/s41467-025-66695-8)
Supplement: Supplementary file 2 — Description of Addtional Supplementary Files [file 41467_2025_66695_MOESM2_ESM.pdf]

Descriptions of Additional Supplementary Files

Supplementary Movie 1: Animation of Model 1

Supplementary Movie 2: Animation of Model 2

Supplementary Movie 3: Animation of Model 3

Supplementary Movie 4: Animation of Model 4

Supplementary Movie 5: Animation of Supplementary Model 1

Supplementary Movie 6: Animation of Supplementary Model 2

Supplementary Movie 7: Animation of Supplementary Model 3

General legend: The models are displayed in 3D-view with the thermo-mechanical model as slices on the right side, and the landscape evolution model on the left. The slices of the thermomechanical model show material colours (see legend in Fig. 9) with temperature contours (100◦C, 350◦C, 550◦C, 1330◦C), and strain weakened areas as dark grey overlay.
